# SARS-CoV-2-Übertragungswege und Implikationen für den Selbst- und Fremdschutz

**DOI:** 10.1007/s00103-021-03389-8

**Published:** 2021-07-29

**Authors:** Djin-Ye Oh, Sindy Böttcher, Stefan Kröger, Max von Kleist

**Affiliations:** 1 Fachgebiet für Influenzaviren und weitere Viren des Respirationstraktes, Robert Koch-Institut, Berlin, Deutschland; 2 Fachgebiet für virale Gastroenteritis- und Hepatitiserreger und Enteroviren, Robert Koch-Institut, Berlin, Deutschland; 3 Fachgebiet für respiratorisch übertragbare Erkrankungen, Robert Koch-Institut, Berlin, Deutschland; 4 Systemmedizin von Infektionskrankheiten, Robert Koch-Institut, Nordufer 20, 13353 Berlin, Deutschland

**Keywords:** COVID-19, Transmission, Überdispersion, Aerosole, Virusausscheidung, COVID-19, Transmission, Overdispersion, Aerosols, Viral shedding

## Abstract

Die weltweite Ausbreitung des Coronavirus SARS-CoV‑2 hat Gesundheits‑, Wirtschafts- und Gesellschaftssysteme massiv in Mitleidenschaft gezogen. Obwohl mittlerweile effektive Impfstoffe zur Verfügung stehen, ist es wahrscheinlich, dass der Erreger endemisch wird und uns noch über Jahre begleitet. Um andere und sich selbst möglichst effektiv vor einer SARS-CoV-2-Infektion zu schützen, ist ein Verständnis der Übertragungswege von größter Wichtigkeit.

In dieser Übersichtsarbeit erläutern wir Übertragungswege im Hinblick auf den Fremd- und Eigenschutz. Darüber hinaus gehen wir auf die Charakteristika der SARS-CoV-2-Übertragung auf Populationsebene ein. Diese Arbeit soll helfen, folgende Fragen anhand der verfügbaren Literatur zu beantworten: Wann und wie lange ist eine infizierte Person kontagiös (ansteckungsfähig)? Wie wird das Virus ausgeschieden? Wie wird das Virus aufgenommen? Wie verbreitet sich das Virus in der Gesellschaft?

Die Mensch-zu-Mensch-Übertragung von SARS-CoV‑2 wird in starkem Maße durch die biologischen Erregereigenschaften, einschließlich der Infektions‑, Replikations- und Ausscheidungskinetik, bestimmt. SARS-CoV‑2 wird hauptsächlich über humane Aerosole übertragen, die von infizierten Personen ausgeschieden werden, auch wenn Erkrankungssymptome (noch) nicht vorliegen. Hieraus resultiert ein relevanter Anteil prä- bzw. asymptomatischer Transmissionen. In geschlossenen Räumen erfolgen Übertragungen besonders effektiv. Die meisten infizierten Personen rufen eine geringe Zahl von Sekundärfällen hervor, während wenige Fälle (sog. Superspreader) zu vielen Folgeinfektionen führen – auf Populationsebene spricht man hier von einer „Überdispersion“. Die besonderen Merkmale von SARS-CoV‑2 (asymptomatische Aerosolübertragung und Überdispersion) machen die Pandemie schwer kontrollierbar.

## Abstract (en)

The global spread of the coronavirus SARS-CoV‑2 has massively impacted health, economic, and social systems. Although effective vaccines are now available, it is likely that this pathogen will become endemic and stay with us for years. In order to most effectively protect others and oneself from SARS-CoV‑2 infection, an understanding of how SARS-CoV‑2 is transmitted is of utmost importance.

In this review paper, we explain transmission routes with an eye towards protecting others and oneself. We also address characteristics of SARS-CoV‑2 transmission in the community. This work will help to clarify the following questions based on the available literature: When and for how long is an infected person contagious? How is the virus excreted? How is the virus taken up? How does the virus spread in society?

Human-to-human transmission of SARS-CoV‑2 is strongly determined by pathogen molecular characteristics as well as the kinetics of replication, shedding, and infection. SARS-CoV‑2 is transmitted primarily via human aerosols, which infected persons can excrete even if symptoms of the disease are not (yet) present. Most infected people cause only a few secondary cases, whereas a few cases (so-called super-spreaders) cause a high number of secondary infections – at the population level one speaks of a so-called “overdispersion.” These special characteristics of SARS-CoV‑2 (asymptomatic aerosol transmission and overdispersion) make the pandemic difficult to control.

## Hintergrund

Die SARS-CoV-2-Pandemie verbreitet sich nunmehr seit über einem Jahr weltweit mit über 180 Mio. bestätigten Fällen und mehr als 3,8 Mio. Menschen, die bis Anfang Juni 2021 unmittelbar an oder mit dem Virus verstorben sind [1]. Mittlerweile sind mehrere zum Teil hocheffektive Impfstoffe in Deutschland zugelassen und werden gemäß der von der Ständigen Impfkommission (STIKO) festgelegten Priorisierung verabreicht [2, 3]. Unter anderem aufgrund von Produktionsengpässen ist die derzeitige Impfkapazität noch begrenzt und die Impfraten liegen unter denen anderer Länder, wie beispielsweise Israel oder den USA, sodass noch nicht klar abschätzbar ist, wann eine breite und wirksame Populationsimmunität erreicht sein wird. Neben der aktiven Immunisierung stehen gegen das Virus gerichtete Biologika (monoklonale Antikörper) für die passive Immunisierung und die Therapie zur Verfügung, da sie bei frühzeitiger Verabreichung den Infektionsverlauf verkürzen und die Krankheitsschwere verringern können [4, 5]. Diese Wirkstoffe zielen alle auf das gleiche molekulare Zielprotein ab (das Spikeglykoprotein), ebenso wie die Immunantwort, welche durch die in Deutschland verfügbaren Impfstoffe ausgelöst wird. In diesem Kontext ist der derzeitig beobachtete Anstieg neuartiger Virusvarianten mit Fluchtmutationen im Spikeprotein mit äußerster Sorge zu betrachten [6–8]. Trotz erheblicher Fortschritte bei der Impfstoffentwicklung und -produktion gehen einige Modellrechnungen davon aus, dass nichtpharmazeutische Maßnahmen wie die AHA + L-Regeln (Einhalten von **A**bstands- und **H**ygieneregeln, das Tragen von (**A**lltags‑)Masken sowie **L**üften) bis ins Jahr 2022 notwendig sein könnten und dass endemische Übertragungen auch noch in den Folgejahren stattfinden werden [9].

Als zentrales Werkzeug jedes SARS-CoV-2-Selbstschutzes ist daher die Vermeidung von infektionsrelevanten Virusexpositionen absolut essenziell. Neben Aktivitäten des Selbstschutzes gibt es Maßnahmen des Fremdschutzes wie die Quarantäne exponierter und potenziell infizierter Personen und die Isolation von symptomatischen oder nachweislich infizierten Individuen [10–12]. Diese beiden Maßnahmen zielen auf die Risikovermeidung der Ansteckung bzw. Übertragung ab.

Dieser Beitrag gibt einen orientierenden Überblick zum derzeitigen Wissensstand zu SARS-CoV-2-Übertragungswegen. Er soll dazu beitragen, potenziell infektionsrelevante Situationen zu erkennen und zu vermeiden, und beleuchtet die derzeitigen Maßnahmen des Fremd- und Selbstschutzes. Im Folgenden werden wir drei Aspekte der Virusübertragung darstellen: (i) Auf Seite des Überträgers betrachten wir zunächst die Grundlagen der Virusreplikation, aus der sich wesentliche Merkmale der Ausscheidungskinetik von SARS-CoV‑2 direkt ableiten lassen. Die Betrachtung dieses Aspektes soll dabei helfen, Maßnahmen des Fremdschutzes besser einordnen zu können. (ii) Auf Seite der exponierten Person betrachten wir die relevanten Aufnahmewege für infektiöse Viruspartikel, was die Einordnung von Selbstschutzmaßnahmen erleichtern soll. Zuletzt wenden wir uns (iii) dem Übertragungsgeschehen auf Populationsebene zu.

## Fremdschutz: Virusreplikation und Ausscheidungskinetik

### Virusreplikation

Wie auch bei anderen Viren erfordert die Vermehrung von SARS-CoV‑2 die Replikation in geeigneten Wirtszellen. Als vorrangig humanpathogenes Virus befällt SARS-CoV‑2 typischerweise menschliche Zielzellen, welche so umprogrammiert werden, dass sie große Mengen neuer Viruspartikel produzieren. Der Zelltropismus (Art der Zellen, in denen eine Vermehrung erfolgen kann) hängt entscheidend davon ab, ob an der Zelloberfläche die Rezeptoren exprimiert sind, die das Virus benötigt, um in die Zelle zu gelangen. SARS-CoV‑2 nutzt hierfür den Angiotensin-konvertierendes-Enzym(ACE)-2-Rezeptor; unterstützt wird der Zelleintritt durch die zelluläre transmembrane Serinprotease 2 (TMPRSS2; [13]). In der Nasenschleimhaut werden sowohl ACE‑2 als auch TMPRSS2 auf hohem Niveau exprimiert, was die effiziente Vermehrung des Virus in den oberen Atemwegen bedingt [14]. Ein weiterer wichtiger Faktor für die Virusvermehrung ist die Verfügbarkeit von Nukleotidbausteinen für die effiziente Replikation des viralen Erbmaterials [15]. Letztere finden sich insbesondere in sich teilenden Zellen (z. B. Epithelzellen; [14]).

Anhand der ersten neun in Deutschland diagnostizierten Patienten, die in München stationär überwacht wurden, erfolgte bereits zu Beginn der Pandemie die umfassende Untersuchung der Ausscheidungskinetik von SARS-CoV‑2 im Menschen. Hierbei wurde gezeigt, dass bei Patienten mit milder bis moderater Krankheitsschwere SARS-CoV‑2 insbesondere in den oberen und unteren Atemwegen sowie im Stuhl nachweisbar ist [16]. Die Virusanzucht im Labor, die als Proxy für Kontagiosität (Ansteckungsfähigkeit) betrachtet wird, war aus Atemwegssekreten ohne viel Aufwand möglich.

Aufgrund dieser und vieler vergleichbarer Studien gilt es als gesichert, dass atemwegsassoziierte Übertragungen die Hauptrolle für die epidemische Ausbreitung von SARS-CoV‑2 spielen [17–19]. Da ein Mund-Nasen-Schutz oder eine Mund-Nasen-Bedeckung die Verteilung virushaltiger Tröpfchen verringern kann, wurden diese zunächst zum Fremdschutz in geschlossenen Gebäuden und Transportmitteln eingeführt. Retrospektive, korrelative Analysen bestätigten den Nutzen dieser Maßnahme [20, 21]. Nachfolgende Studien zeigten, dass nicht nur größere Tröpfchen, sondern auch kleinere virushaltige Tröpfchenpartikel (Aerosole), die beim Atmen, Sprechen, Singen, Niesen oder Husten ausgeschieden werden, in erheblichem Maße zur Virusausbreitung beitragen [22, 23].

### Zeitlicher Verlauf der Virusausscheidung

Anhand der ersten in Deutschland aufgetretenen Fälle wurde schon zu Anfang der Pandemie die Viruskinetik bei mild verlaufenden SARS-CoV-2-Infektionen mit besonderem Augenmerk auf die Quantifizierung und die Anzüchtbarkeit (als Maß für das Vorhandensein infektiöser Viruspartikel) untersucht [16]. Später durchgeführte Studien in größeren Patientenkollektiven [18, 19, 24] lieferten noch genauere Einblicke in die Infektionskinetik und den zeitlichen Verlauf der Kontagiosität. Hierbei fanden sich große interindividuelle Unterschiede in der Ausscheidungskinetik: Im Vergleich zu mild-symptomatischen oder asymptomatischen Patienten scheiden beispielsweise Patienten mit schweren oder kritischen Krankheitsverläufen deutlich länger infektiöse Viren aus [19, 25, 26]. Gleiches gilt für immunkompromittierte Individuen [27–30]. Neben diesen interindividuellen Differenzen der Ausscheidungskinetik werden die gemessenen Viruslasten aber auch von präanalytischen Faktoren mitbestimmt, wie beispielsweise der Art von Probenentnahme, -transport und -aufbereitung.

Dennoch erlaubten diese Studien es, die Infektiosität des aus den Atemwegen gewonnenen Probenmaterials sowohl mit der Viruslast als auch mit der Dauer der Infektion zu korrelieren [10, 11]: Höhere Viruslasten im Probenmaterial gehen dabei mit leichterer Anzüchtbarkeit in der Zellkultur einher als niedrige Viruslasten. Des Weiteren ist die Anzuchtwahrscheinlichkeit umso höher, je früher im Krankheitsverlauf eine Probe gewonnen wurde. Dies kann über die im Verlauf der Erkrankung zunehmende Wirksamkeit der Immunantwort, in deren Zuge der Anteil neutralisierter (d. h. mit Antikörpern besetzter) Viruspartikel zunimmt, plausibel erklärt werden; umgekehrt scheiden immunkompromittierte Individuen (insbesondere solche mit B‑Zell-Defekt) infektiöse Viren länger aus [27–30].

Nach Infektion eines neuen Wirts vermehrt sich SARS-CoV‑2 typischerweise zunächst in den Epithelzellen der oberen Atemwege, sodass es dort zu einem Anstieg der Viruslast und zur Ausscheidung infektiöser Viren kommt. Dies geschieht bereits während der Inkubationszeit (präsymptomatische Phase) also vor dem Auftreten erster Krankheitssymptome. Die Viruslast in den oberen Atemwegen nimmt zu, bis sie einen maximalen Wert erreicht. Die derzeitige Studienlage deutet darauf hin, dass dieser maximale Wert zeitlich in etwa mit dem Auftreten erster Krankheitssymptome zusammenfällt [16, 29, 31, 32]. Anschließend erfolgt ein kontinuierlicher Abfall der Viruslast in den Sekreten der oberen Atemwege, während die Viruslast in den unteren Atemwegen ihren Spitzenwert im Allgemeinen später erreicht und generell langsamer abfällt. Dementsprechend weist eine virologische Diagnostik unter Verwendung von Probenmaterialien der unteren Atemwege, wie z. B. Sputum (Hustenauswurf), oftmals länger positive Untersuchungsergebnisse auf als beispielsweise Abstriche der oberen Atemwege [12, 16].

Die Ergebnisse zahlreicher Kontaktpersonenuntersuchungen in Haushalten zur SARS-CoV-2-Übertragung weisen darauf hin, dass die Ansteckungsfähigkeit in der Zeit um den Symptombeginn herum besonders groß ist [32, 33]. Mehr als die Hälfte aller Übertragungen geht von prä- bzw. asymptomatischen Personen aus [31, 32, 34–37]. Das Übertragungsrisiko, das von infizierten Personen ausgeht, die keine Symptome zeigen (asymptomatische Infizierte), lässt sich nur schwer abschätzen, denn häufig liegt hier eine Untererfassung vor. Ein relevanter Anteil der Ansteckungen geht von infektiösen Personen innerhalb von 1–2 Tagen vor deren Symptombeginn aus [32, 38]. Das Zeitintervall zwischen Infektion und Beginn der Kontagiosität unterliegt großer interindividueller Variation und ist bislang nicht klar bestimmt worden. Einzelbeobachtungen deuten darauf hin, dass dieses Zeitfenster in manchen Fällen sehr kurz sein kann und dass Transmissionen bereits am Tag nach Infektion erfolgen können. Der hohe Anteil prä-/asymptomatischer SARS-CoV-2-Transmissionen gilt als einer der Hauptfaktoren, die die weltweite Ausbreitung des Erregers binnen kurzer Zeit ermöglicht haben [39].

Die Senkung des Übertragungsrisikos und die Eindämmung der epidemischen Ausbreitung von SARS-CoV‑2 erfordern die möglichst rasche Isolierung positiv getesteter Personen ebenso wie die Identifikation und frühzeitige Quarantäne enger Kontaktpersonen. Auf der Grundlage klinischer Studien zur Viruskinetik konnten am Robert Koch-Institut (RKI) mathematische Modelle entwickelt werden (https://covidstrategycalculator.github.io/), die für die Festlegung von Quarantäne- und Isolationsdauer herangezogen wurden [10, 11]. Darüber hinaus erfolgen Maßnahmen, welche die Übertragung durch (noch) nicht erkannte prä- oder asymptomatische Infizierte verhindern: das Abstandhalten zu anderen Personen, das Einhalten von Hygieneregeln, das Tragen von (Alltags‑)Masken sowie Lüften (AHA + L-Regel).

Seit Ende 2020 sind besorgniserregende Virusvarianten beschrieben, für die eine höhere Ansteckungsfähigkeit vermutet wird oder bereits erwiesen ist [8]. Die zugrunde liegenden Mechanismen sind bislang nicht eindeutig geklärt. Diskutiert wird z. B. im Fall der besorgniserregenden Variante B.1.1.7 (sog. Alpha-Variante) eine differente Viruskinetik, bei der übertragungsrelevante Virusmengen über eine längere Zeitdauer ausgeschieden werden, sodass die Kontagiosität insgesamt erhöht ist [40]. Des Weiteren diskutiert man neben höheren Viruslasten auch, dass eine geringere infektiöse Dosis zur Etablierung einer Infektion benötigt werden könnte [41–43].

## Selbstschutz: Aufnahme von Viruspartikeln bei der Mensch-zu-Mensch-Übertragung

Der wichtigste Übertragungsweg für SARS-CoV‑2 ist die respiratorische Aufnahme virushaltiger Partikel unterschiedlicher Größe, die aus dem Respirationstrakt infizierter Personen freigesetzt werden, wenn diese husten, niesen, schreien, singen, sprechen oder atmen [22, 23, 44]. Diese virushaltigen Partikel „landen“ auf den Schleimhäuten von Kontaktpersonen oder werden von diesen eingeatmet, woraus eine Folgeinfektion resultieren kann (besprochen in [45]; [22, 23, 44]). Partikel eines Durchmessers über ~ 100 µm werden häufig als „Tröpfchen“ (Droplets) bezeichnet [45]. Nach Emission folgen sie einer ballistischen Flugbahn und sinken im Abstand von 1–2 m von der emittierenden Person zu Boden [46]. Aufgrund ihrer Größe werden sie im Allgemeinen nicht eingeatmet, können sich aber auf der Schleimhaut einer exponierten Person absetzen und so eine Infektion vermitteln [45]. Partikel eines Durchmessers unter ~100 µm werden oft als „Aerosole“ bezeichnet und sind im Gegensatz zu den größeren Tröpfchen einatembar [45]. Sie können über Minuten oder gar Stunden in der Luft schweben, vor allem bei geringer Temperatur und niedriger Luftfeuchtigkeit [22]. Ihre höchste Konzentration erreichen Aerosole in der unmittelbaren Nähe der emittierenden Person, sie können jedoch weite Strecken zurücklegen und Transmissionen über größere Distanzen vermitteln.

Während beim Husten und Niesen besonders viele Tröpfchen entstehen [47], werden beim Atmen, Sprechen, Schreien und Singen virushaltige Aerosole in großer Menge ausgeschieden [44, 45, 47–50]. Bei geringem Abstand, also weniger als etwa 1,5 m von einer infizierten Person, besteht eine erhöhte Wahrscheinlichkeit der Exposition gegenüber infektiösen Partikeln jeglicher Größe [51]. Historisch wurde die Abgrenzung zwischen Tröpfchen und Aerosolen häufig anhand eines Partikeldurchmesserwertes von 5 µm vorgenommen, die diesem Wert zugrunde liegende medizinische bzw. physikalische Rationale ist allerdings unklar. Mittlerweile akzeptierter ist der Grenzwert von ~100 µm, oberhalb dessen Partikel im Allgemeinen nicht mehr eingeatmet werden können [45, 46, 52].

Ob ein Krankheitserreger vorwiegend über Tröpfchen oder Aerosole übertragen wird, hängt von Wirtsfaktoren, Pathogenfaktoren sowie deren Zusammenspiel ab [45]. Die genaue Bestimmung der Hauptübertragungswege ist keineswegs trivial. Insbesondere wenn Aerosolübertragung über kurze Distanz erfolgt (dies ist häufig der Fall, da die Aerosolkonzentration in unmittelbarer Nähe der emittierenden Person am höchsten ist), ist das epidemiologische Erscheinungsbild von dem einer Tröpfcheninfektion oft nicht klar unterscheidbar [22, 44, 47–51].

Weder für SARS-COV‑2 noch für andere respiratorische Viren ist abschließend geklärt, welcher Transmissionsmechanismus (Tröpfchen oder Aerosole) prädominiert. Jedoch gibt es eine Fülle an Hinweisen, dass SARS-CoV‑2 in erster Linie durch Aerosole übertragen wird, vor allem über kurze Distanzen (< 1,5 m), aber insbesondere in Innenräumen auch über weitere Distanzen [45, 52–56].

Virushaltige Aerosole können sich in Innenräumen anreichern, insbesondere wenn diese unzureichend belüftet sind, woraus ein besonders hohes Infektionsrisiko resultiert. Dieses Risiko nimmt zu, je dichter die Raumbesetzung und je länger die Aufenthaltsdauer ist. Das Übertragungsrisiko erhöht sich außerdem, wenn Tätigkeiten mit größerer Aerosolausscheidung stattfinden (z. B. Schreien, Singen, körperliche Arbeit, Sport) und auch wenn die virusexponierten Personen mehr Atemarbeit leisten. Dementsprechend sind große Ausbrüche in folgenden Zusammenhängen beschrieben: Chorproben [57], Bars [58], Fitnessstudios [59] und Fleisch verarbeitende Betriebe [60].

Durch regelmäßigen, effektiven Luftaustausch kann die Aerosolkonzentration in einem Innenraum gemindert werden [61, 62]. Geeignete Luftreiniger, die Aerosole mittels spezieller Filter aus der Luft entfernen, können hierzu ebenfalls beitragen [63, 64]. Darüber hinaus können Mund-Nasen-Schutzmasken bzw. angepasste FFP2-Masken die Aerosolaufnahme im Sinne des Selbstschutzes verringern [44].

In Außenbereichen sind Übertragungen von SARS-CoV‑2 deutlich seltener als in Innenräumen zu beobachten, insbesondere wenn starke Luftbewegung besteht und Mindestabstände gewahrt werden [65]. Die Übertragung durch kontaminierte Oberflächen ist insbesondere in der unmittelbaren Umgebung der infektiösen Person zwar nicht auszuschließen, spielt jedoch im Gesamtübertragungsgeschehen vermutlich eine untergeordnete Rolle [66]. Das Risiko einer Kontaktübertragung kann durch Händewaschen und Oberflächenreinigung gemindert werden.

## Übertragungswege in der Gesellschaft

Die Wahrscheinlichkeit, sich mit SARS-CoV‑2 zu infizieren, hängt zunächst von der Wahrscheinlichkeit einer Exposition mit virushaltigem Material bei Kontakt zu kontagiösen Personen ab. Die Wahrscheinlichkeit, sich bei einer solchen Exposition mit SARS-CoV‑2 zu infizieren, wird wesentlich von der Gesamtmenge des eingeatmeten Virus bestimmt. Einflussfaktoren für Übertragungen in Innenräumen sind daher die Belüftung, die Anzahl der anwesenden (infizierten) Personen, die Dauer des Aufenthalts und die Art der ausgeführten Aktivitäten [67]. Grundsätzlich kann jegliche Aktivität, die ein Risiko der Einatmung von Viruspartikeln in sich birgt, zum Infektionsgeschehen beitragen.

### Säulen und Treiber des Infektionsgeschehens

Für SARS-CoV‑2 wird angenommen, dass etwa 10 % der Fälle für 80 % der Infektionen verantwortlich sein könnten [68, 69], d. h., es wird bei der Übertragung von SARS-CoV‑2 von einer sogenannten Überdispersion ausgegangen [70–73]. Dies bedeutet, dass die meisten infizierten Personen eine geringe Zahl von Sekundärfällen hervorrufen, während wenige Fälle zu vielen Folgeinfektionen führen. Derartige „Superspreading-Events“ sind zwar selten, führen aber zu vielen Folgeinfektionen [74]. Erstere führen dazu, dass die Pandemie sich stetig fortsetzt („Säulen“), während Letztere die Pandemie „anfachen“ („Treiber“). Zwischen „Säulen“ und „Treibern“ bestehen in der Realität fließende Übergänge.

Die „Säulen“ der Pandemie finden sich in Settings, in denen enge, lang andauernde Kontakte bestehen, wie beispielsweise in Haushalten. Die Wahrscheinlichkeit der Weitergabe einer Infektion im Haushalt, wird im Allgemeinen auf etwa 20 % geschätzt [75, 76]. Eine koreanische Studie mit 5706 untersuchten Haushalten legt anhand von Kontaktverfolgungsdaten nahe, dass in etwa 57 % der Folgeinfektionen im häuslichen Umfeld geschehen [77]. Allerdings sind in Kontaktnachverfolgungsdatensätzen Übertragungswege naturgemäß unterrepräsentiert, bei denen die übertragende Person oder die Kontaktpersonen einander nicht kennen. Durch das Zusammenführen von Daten der molekularen Surveillance (Virusgenomdaten aus der Gesamtgenomsequenzierung) mit epidemiologischen Daten kann ein erheblich größerer Anteil an Übertragungswegen aufgeschlüsselt werden, wie dies kürzlich anhand einer australischen Studie illustriert worden ist [78]. In Deutschland erlaubt die integrierte molekulare Surveillance von SARS-CoV‑2 derzeit einen differenzierteren Einblick in die Ausbreitungs- und Transmissionsdynamiken von SARS-CoV‑2 [8].

Als „Treiber“ der Pandemie lassen sich Veranstaltungen in Innenräumen betrachten, bei denen viele Menschen über längere Zeit auf engem Raum zusammenkommen. Beispiele sind Feiern [79], private Reisen [80, 81], gemeinsame Freizeitaktivitäten sowie Arbeitstreffen und Konferenzen [57, 58, 82].

Der Beitrag von Kindern zum Pandemiegeschehen wird kontrovers diskutiert. Genauso wie Erwachsene können sich auch Kinder mit SARS-CoV‑2 infizieren, sie zeigen jedoch häufiger ein asymptomatisches oder mildes klinisches Bild [83–85]. Allerdings ist das Ausmaß möglicher Spätfolgen einer SARS-CoV-2-Infektion (Long Covid) im Kindes- und Jugendalter bislang unzureichend charakterisiert [86–88]. Darüber hinaus können auch asymptomatisch infizierte Kinder, die zwar sehr seltene, aber schwere und häufig intensivpflichtige Folgeerkrankung entwickeln (PIMS – „paediatric inflammatory multisystem syndrome“; MIS-C – „multisystem inflammatory syndrome in children“), deren Fallsterblichkeitsrate auf 1,4–3 % geschätzt wird [89–91]. Der milde bzw. asymptomatische Verlauf im Kindesalter birgt die Gefahr einer Unterschätzung pädiatrischer Fallzahlen, insbesondere wenn eine „symptomzentrierte“ Teststrategie zum Einsatz kommt, welche die SARS-CoV-2-Testungen auf symptomatische Patienten oder Kontaktpersonen fokussiert, wie es insbesondere zu Beginn der Pandemie der Fall war [92]. Auch wenn das von Kindern ausgehende Transmissionsrisiko noch immer nicht abschließend quantifiziert werden kann, ist es mittlerweile unbestritten, dass SARS-CoV-2-infizierte Kinder das Virus weitergeben können. Für Kinder > 10 Jahre wurde dies bereits früh in der Pandemie gezeigt [77, 93–95].

Untersuchungen zu Ausbrüchen in Kita- und Schulsettings zeigen eine Abhängigkeit vom Infektionsgeschehen in der Allgemeinbevölkerung, aber es wird ihnen keine überragende Treiberfunktion zugeschrieben, wie dies beispielsweise bei Influenza der Fall ist [96, 97]. Da es in Schulen zu zahlreichen, bezüglich Dauer und Nähe intensiven Kontakten in Innenräumen kommt, ist davon auszugehen, dass sie, in Abhängigkeit vom Infektionsgeschehen in der Allgemeinbevölkerung, relevant zum Pandemiegeschehen beitragen können, auch wenn von jüngeren Kindern möglicherweise ein reduziertes Transmissionsrisiko ausgeht [77, 98–100]. Deshalb ist es wichtig, dass in Schulen und Betreuungseinrichtungen geeignete Konzepte zur Infektionsprävention eingesetzt werden [101, 102]. Wenn diese konsequent umgesetzt werden, ist davon auszugehen, dass die Durchführung von Präsenzunterricht möglich ist, ohne dass ein erhöhtes Infektionsrisiko für Schulkinder besteht [103].

### Maßnahmen

Während sich die Verbreitung von SARS-CoV‑2 im häuslichen Umfeld schwer kontrollieren lässt, können Settings mit Superspreading-Potenzial gezielt adressiert werden, um die Pandemie entscheidend zu verlangsamen. Hierzu eignen sich Maßnahmen wie die vermehrte Arbeit im Homeoffice, die Vermeidung von Massenveranstaltungen, Kontaktreduktionen und an Orten, wo eine Exposition nicht vermeidbar ist (z. B. in Schulen, Pflegeeinrichtungen und generell Innenräumen), das Tragen von Schutzmasken und die Umsetzung von Lüftungskonzepten, welche auch den Einsatz geeigneter Luftreiniger einschließen können. Darüber hinaus sind Teststrategien, z. B. regelmäßige, gepoolte Polymerasekettenreaktion(PCR)-Testungen von festen Gruppen (z. B. Kita, Schule), zusätzliche effektive Maßnahmen [104, 105]. Korrelative Analysen von Eindämmungsmaßnahmen während der ersten SARS-CoV-2-Welle bestätigen, dass diese eine Reduktion der effektiven Reproduktionszahl nach sich ziehen und somit wirksame Werkzeuge der Pandemiekontrolle darstellen [21, 106].

## Fazit

Das Coronavirus SARS-CoV‑2 breitet sich weiterhin aus. Obwohl effektive Impfstoffe zur Verfügung stehen, sind diese global ungleich verteilt, sodass es wahrscheinlich ist, dass der Erreger noch über Jahre hinweg eine globale Bedrohung darstellt. Um andere und sich selbst möglichst effektiv vor einer SARS-CoV-2-Infektion zu schützen, ist daher ein Verständnis der Übertragungswege von SARS-CoV‑2 von größter Wichtigkeit.

Die SARS-CoV‑2 Übertragungswege werden in starkem Maße durch die biologischen Erregereigenschaften bestimmt. Das Virus wird hauptsächlich über humane Aerosole übertragen, die auch zu einem erheblichen Anteil von prä- bzw. asymptomatisch Infizierten Personen ausgeschieden werden, sodass AHA + L-Regeln weiterhin eine effektive Maßnahme des Fremd- und Eigenschutzes darstellen. Superspreading-Events gelten als Pandemietreiber, wohingegen die Rolle von Kindern in der Pandemie nach wie vor nicht abschließend geklärt ist.
